# Rheological effects of hypertonic saline and sodium bicarbonate solutions on cystic fibrosis sputum in vitro

**DOI:** 10.1186/s12890-021-01599-z

**Published:** 2021-07-12

**Authors:** Mária Budai-Szűcs, Szilvia Berkó, Anita Kovács, Pongsiri Jaikumpun, Rita Ambrus, Adrien Halász, Piroska Szabó-Révész, Erzsébet Csányi, Ákos Zsembery

**Affiliations:** 1 Institute of Pharmaceutical Technology and Regulatory Affairs, University of Szeged, Szeged, Hungary; 2 Department of Oral Biology, Semmelweis University, Budapest, Hungary; 3 National Korányi Institute for Pulmonology, Budapest, Hungary

**Keywords:** Cystic fibrosis, Rheology, Hypertonic salt solutions, Bicarbonate, In vitro treatment

## Abstract

**Background:**

Cystic fibrosis (CF) is a life-threatening multiorgan genetic disease, particularly affecting the lungs, where recurrent infections are the main cause of reduced life expectancy. In CF, mutations in the gene encoding the cystic fibrosis transmembrane conductance regulator (CFTR) protein impair transepithelial electrolyte and water transport, resulting in airway dehydration, and a thickening of the mucus associated with abnormal viscoelastic properties. Our aim was to develop a rheological method to assess the effects of hypertonic saline (NaCl) and NaHCO_3_ on CF sputum viscoelasticity in vitro, and to identify the critical steps in sample preparation and in the rheological measurements.

**Methods:**

Sputum samples were mixed with hypertonic salt solutions in vitro in a ratio of either 10:4 or 10:1. Distilled water was applied as a reference treatment. The rheological properties of sputum from CF patients, and the effects of these in vitro treatments, were studied with a rheometer at constant frequency and strain, followed by frequency sweep tests, where storage modulus (G′), loss modulus (G″) and loss factor were determined.

**Results:**

We identified three distinct categories of sputum: (i) highly elastic (G′ > 100,000 Pa), (ii) elastic (100,000 Pa > G′ > 1000 Pa), and (iii) viscoelastic (G′ < 1000). At the higher additive ratio (10:4), all of the added solutions were found to significantly reduce the gel strength of the sputum, but the most pronounced changes were observed with NaHCO_3_ (p < 0.001). Samples with high elasticity exhibited the greatest changes while, for less elastic samples, a weakening of the gel structure was observed when they were treated with water or NaHCO_3_, but not with NaCl. For the viscoelastic samples, the additives did not cause significant changes in the parameters. When the lower additive ratio (10:1) was used, the mean values of the rheological parameters usually decreased, but the changes were not statistically significant.

**Conclusion:**

Based on the rheological properties of the initial sputum samples, we can predict with some confidence the treatment efficacy of each of the alternative additives. The marked differences between the three categories suggest that it is advisable to evaluate each sample individually using a rheological approach such as that described here.

**Supplementary Information:**

The online version contains supplementary material available at 10.1186/s12890-021-01599-z.

## Introduction

Cystic fibrosis (CF) is one of the most common life-threatening genetic diseases. It affects many organs, including the lungs, where viscous mucus clogs the airways and recurrent infections shorten the patients’ lifespan [1]. In CF, transepithelial Cl^−^ and HCO_3_^−^ transport is impaired due to mutations in the gene encoding the cystic fibrosis transmembrane conductance regulator (CFTR) protein, which functions as a cAMP/PKA-regulated epithelial anion channel. Since, in addition, CFTR inhibits the activity of the epithelial Na^+^ channel (ENaC), there is also hyper-reabsorption of Na^+^ in CF airways. The reduced concentrations of Na^+^ and Cl^−^ in the airway surface liquid (ASL) cause water depletion, resulting in airway dehydration, and a thickening of the mucus associated with abnormal viscoelastic properties and osmotic pressures. The rheological properties of the mucus strongly influence the effectiveness of mucus clearance and cough [2–4]. Changes in these properties significantly reduce mucus clearance in CF and thus contribute to the colonization of the airways by bacteria and the development of recurrent infections [5, 6].

Recent studies have focused on impaired transepithelial HCO_3_^−^ transport [4, 7, 8] because it has a key role in maintaining the normal pH of the ASL. Low levels of HCO_3_^−^ lead to acidic ASL pH, which favours bacterial growth and compromises immune defence. However, HCO_3_^−^ concentrations also determine the physiological properties of mucins. Negatively-charged amino acid residues in newly-formed mucin molecules are bound to Ca^2+^ and protons, which must be removed to ensure mucin expansion and hydration during the formation of the extracellular mucus gel. Physiologically, this process is facilitated by HCO_3_^−^, which can form complexes with Ca^2+^ and protons [9, 10].

The gel structure of mucus also depends on various interactions within and between the mucin molecules. Major interactions include disulphide, hydrogen and ionic bonds, as well as physical entanglements [11]. In dehydrated ASL, the high concentration of mucin molecules increases the number of crosslinks, resulting in highly elastic sputum in CF patients. Mucolytic agents are designed to break these crosslinks, thereby improving the rheological properties of the ASL. Previous studies have shown that mucus viscosity can be reduced by the administration of a hypertonic NaCl solution, which disrupts the ionic interactions between the mucin chains [11]. In addition, when a hypertonic salt solution (HS) is used, fluid moves by osmosis from the interstitial space to the airways. This further weakens the gel structure, increases the volume of the ASL and stimulates mucociliary clearance (MCC) [12, 13].

The rheological properties of human sputum can be a useful indicator of disease severity or progression as well as treatment efficacy. In order to assess sputum viscoelasticity in clinical practice, a comprehensive understanding of practical rheology is required, including sample preparation, the choice of parameter settings, and knowledge of the external factors influencing the measurements.

Our specific aim in this study was to develop a rheological method for assessing the effects of hypertonic saline (NaCl) and NaHCO_3_ on CF sputum viscoelasticity in vitro.

## Materials and methods

### Materials

Hypertonic solutions, 300 mmol/L sodium chloride (Ph.Eur, Hungaropharma Zrt, Budapest, Hungary) and 300 mmol/L sodium bicarbonate (Ph.Eur, Hungaropharma Zrt, Budapest, Hungary), were freshly prepared.

Sputum samples of CF patients (both male and female over 18 age) were spontaneously produced and collected in the National Korányi Institute for Pulmonology (Budapest, Hungary). Patients were in a stable condition and had received neither hypertonic saline nor dornase alfa therapy for 8 h before the sputum collection. Sputum samples were immediately frozen and stored at − 20 °C. Before use, samples were thawed at room temperature. All methods were carried out in accordance with relevant guidelines and regulations. Experimental protocols were approved by both the Ethics Committee of the National Korányi Institute for Pulmonology (registration number: 7/2019) and the Hungarian Medical Research Council (ETT-TUKEB, registration number: IV/8155-3/2020/EKU). Informed consent was obtained from all subjects.

In order to separate the sputum from saliva, samples were centrifuged (Hermle centrifuge) at 11,000 RCF for 10 min [4]. The supernatant saliva was then decanted, and the sputum samples from the same patient pooled and gently stirred for homogenization with a magnetic stirrer at 100 rpm for 10 min. This procedure enabled to analyse a concentrated sputum sample.

Sputum samples were divided into aliquots in order to compare the effects of different treatments on the same samples. Alternative methods (horizontal shaking, ultrasonication, magnetic stirring or gentle manual mixing) were evaluated for mixing the sputum samples with the additives (either water or the hypertonic solutions).

Sputum samples were treated with either distilled water (n = 87) or hypertonic NaCl (n = 74) or NaHCO_3_ (n = 55). The ratio of sputum to additive solution was either 10:4 or 10:1 by volume.

### Rheological methods

The rheological properties of the sputum in CF, and the effects of the in vitro treatments, were studied with a Physica MCR302 rheometer (Anton Paar, Austria). The measuring device was of parallel plate type (diameter 25 mm, gap 0.1 mm). The measurements were carried out at 37 °C, and evaporation was blocked with a cap containing water.

In the first part of the measurement (the resting phase), the structural state of the sputum, and any changes resulting from the treatments, were determined over 30 min at a constant angular frequency of 1 rad/s and a constant strain of 0.4%. Each measurement was performed immediately after mixing the solutions and gentle agitation.

Viscoelastic characteristics were determined by frequency-sweep tests immediately after the resting-phase measurement, using a strain of 0.4%. Storage modulus (G′), loss modulus (G″) and loss factor (tanδ) were determined over an angular frequency range from 0.1 to 100 rad/s. The strain value (0.4%) used in the measurements was within the linear range of the viscoelasticity of the sputum.

We assessed both untreated and treated samples for each sputum specimen. During the development of the methodology, parallel samples from the same patient collected at a given time were used for the comparison of the different treatments.

### Statistical analysis

One-way ANOVA followed by Dunnett’s test, with GraphPad Prism 8.0 software (GraphPad Software Inc., San Diego, USA) were applied for statistical analysis. p < 0.05 was considered to be statistically significant, whereas p ≤ 0.01 and p ≤ 0.001 were considered very and highly significant, respectively.

## Results

In order to analyse the possible breakdown of mucus structure in CF, and to follow the recovery of the sputum samples after their treatment and insertion into the rheometer, a constant oscillation test was applied at low frequency and strain value. This part of the measurement, here denoted the ‘resting phase’, can be beneficial in gaining insight into the time-dependency of the in vitro treatments.

**Fig. 1:**
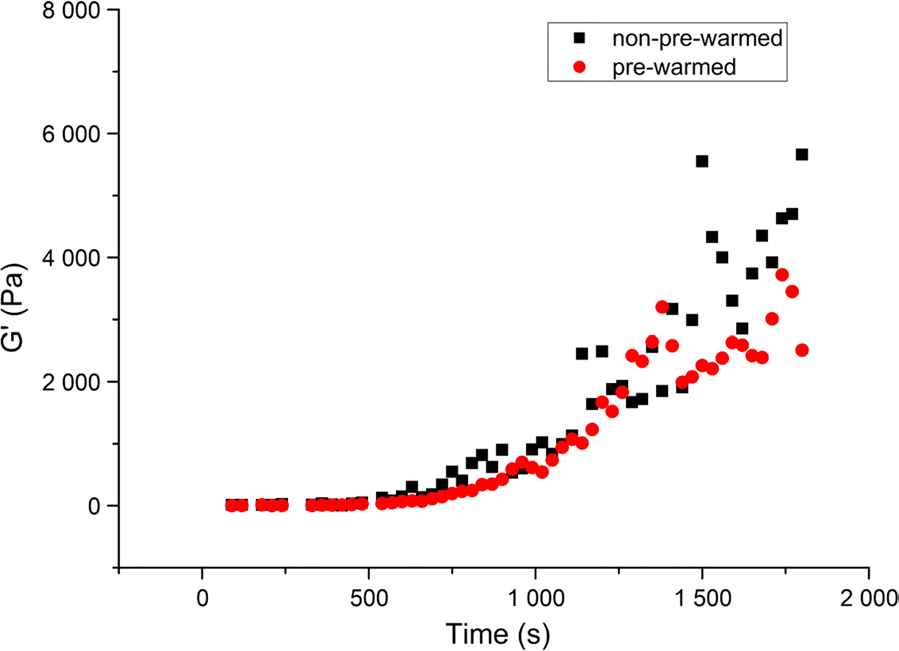
Time-dependent representative changes in the storage modulus (G′) of CF sputum samples during the resting phase of rheological measurements on pre-warmed and non-pre-warmed samples

During initial trials of the methodology, we observed a strong increase in the G′ value, typical of gelation. The samples were routinely protected against evaporation by a blocking cap containing water, so these effects could have been due either to structural breakdown of the sample during insertion or to thermogelation of the sputum, which had been stored at room temperature before the measurements. In order to distinguish between these two possibilities, experiments were performed with sputum samples pre-incubated at 37 °C for 30 min and inserted into the pre-warmed instrument at the same temperature. The resting phase of the pre-warmed sputum showed the same characteristics as those that were not pre-warmed before the measurements (Fig. 1), indicating that the storage temperature did not influence the rheological behaviour of the sputum samples. This result suggests a destruction effect of the installation process on the gel structure of the sputum.

The effects of different types of agitation were also assessed during the development of our in vitro treatment method. The sputum samples were divided into 3 parts: (i) untreated, (ii) mixed with an orbital shaker for 15 min at 450 rpm, and (iii) treated in an ultrasound bath for 5 min.

**Fig. 2:**
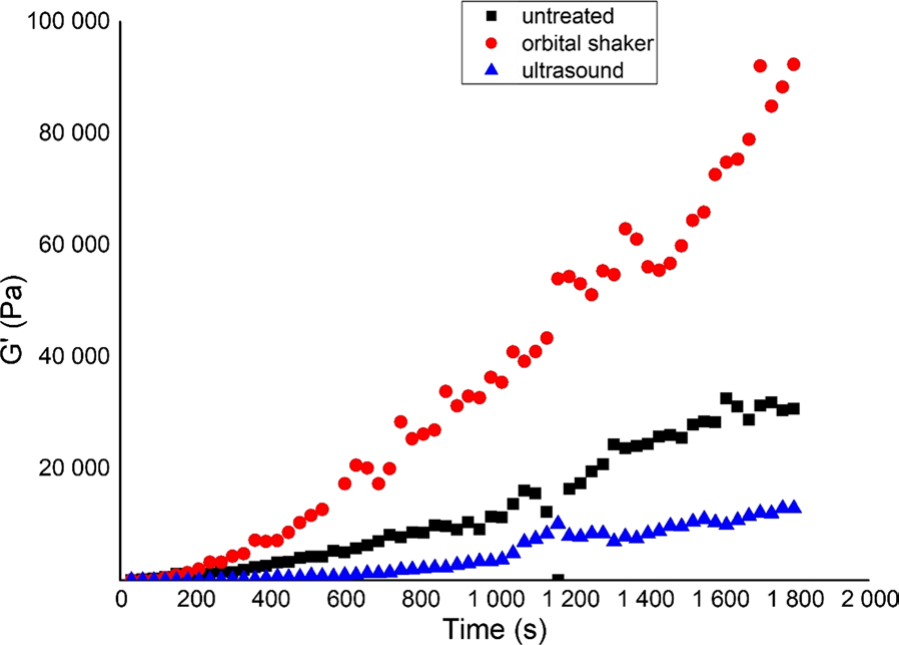
Resting phase of representative sputum samples after different types of agitation

The recovery part of the same sample varied according to the different pre-treatments (agitations) (Fig. 2).

The orbital shaker improved the increase in viscoelastic behaviour during the resting phase, while the US destroyed the structure. Sonication resulted in heating and the precipitation of some components of the sputum, therefore, we discontinued to use this method.

We tried to mimic physiological conditions while performing the in vitro treatments. When hypertonic saline solutions are used by inhalation, no real mixing occurs because the inhaled saline solution is simply sprayed onto the airway surface liquid. Therefore, we applied gentle manual agitation at room temperature for 10 s, followed by the insertion of the mixture into the rheometer and the immediate start of the measurement.

**Fig. 3:**
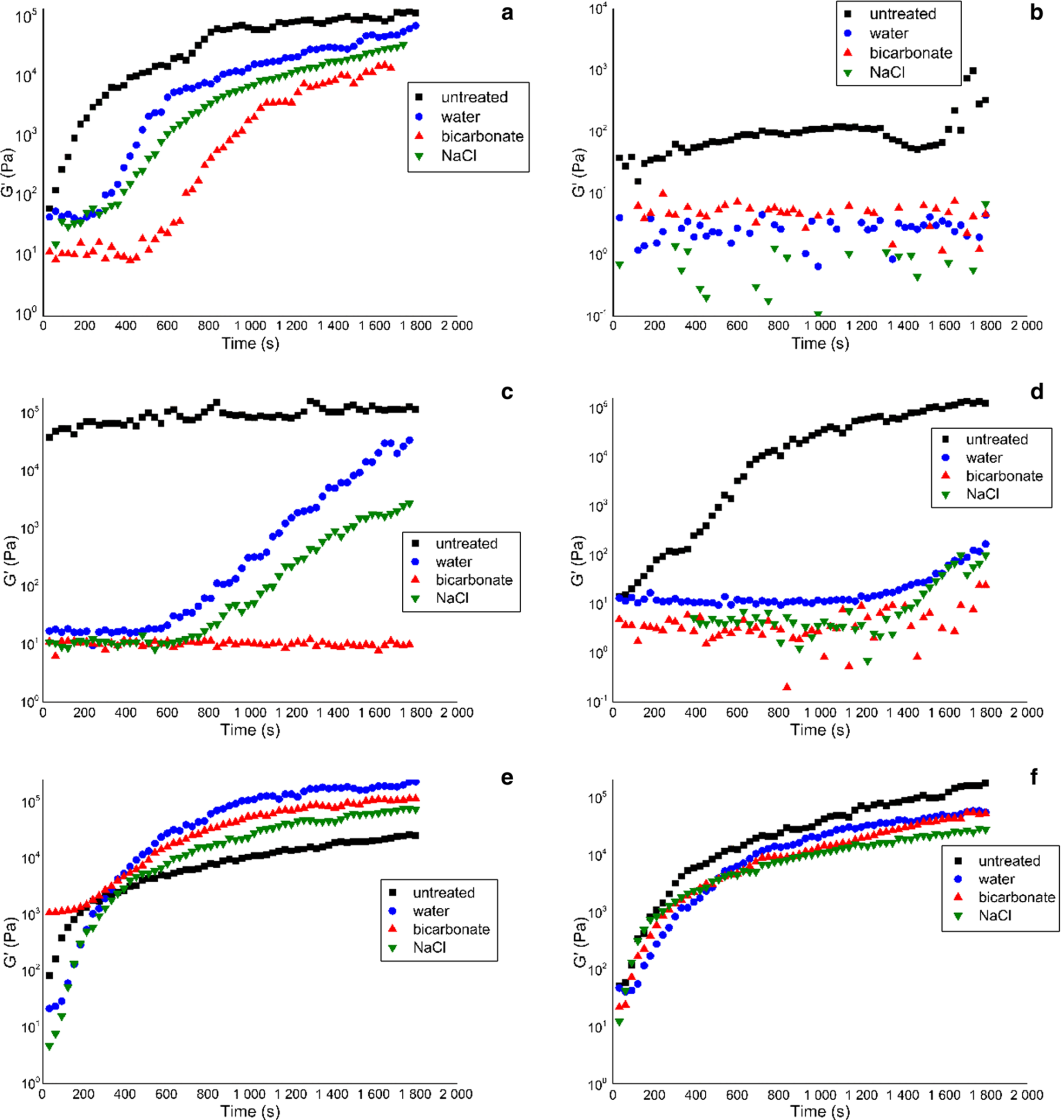
Resting phase of sputum samples after different treatments in the case of different patients

The resting phase was examined for all of the CF sputum samples obtained from the CF patients and subjected either to no treatment or mixing with water, hypertonic NaCl or hypertonic NaHCO3. Our data show that significant gel structure build-up occurred in some samples (Fig. 3).

In many cases the storage modulus G' of the sputum sample increased significantly during the measurements (Fig. 3a, d, e, f), reflecting the recovery of gel structure. In some of those cases, the rate of recovery was significantly altered by the various pre-treatments. In general, the added water or hypertonic solutions slowed down recovery (Fig. 3a, c, d), while in some cases they completely prevented the recovery of the strong gel structure (Fig. 3b). In a few cases, however, there were no significant changes in this parameter (Fig. 3f).

This resting phase is very important because the structure of the sputum can change during insertion into the rheometer, and recovery can be observed even in the untreated samples, suggesting significant adverse effects of the sample insertion procedure.

Mixing with additives and gentle agitation can result in slower recovery, and in some cases the gel structure did not build up again. In order to gain information about the real gel structure and the effect of the pre-treatment and the additives, we considered the strong effects of mixing and installation on the gel structure and assessed the final gel structure, which requires the inclusion of a resting phase in the rheological measurements. In most cases, the additives delayed the recovery of the gel structure, but no significant differences were revealed between the effects of the additives.

The effects of the different additives were evaluated in detail based on frequency-sweep tests. Storage and loss moduli, G′ and G″ respectively, of both untreated and treated sputum samples were compared with each other. The values were interpreted at angular frequencies of 1 and 10 rad/s.

A centrifugation was applied during the sputum samples preparation, which resulted in a concentrated sputum sample [4]. Despite of standard pre-treatment, concentrated sputum samples presented very different gel strengths. The gel strengths of the original sputum samples were very different. We could distinguish 3 categories of sample based on the G′ values of the untreated samples determined at an angular frequency of 1 rad/s in the resting phase of the measurements: (i) highly elastic (G′ > 100,000 Pa), (ii) elastic (100,000 Pa > G′ > 1000 Pa) and (iii) viscoelastic (G′ < 1000) sputum samples. Based on these categories, the efficacy of the treatments was evaluated for all samples by category and the mixing ratio of the sputum and additives: 10:4 (Table 1) and 10:1 (Table 2).

**Table 1:** Rheological parameters of the sputum using a sputum: additive ratio of 10:4

	Untreated	Water		bicarbonate		NaCl	
	Mean (SD)	Mean (SD)	p value	Mean (SD)	p value	Mean (SD)	p value
*For all samples*							
G′ (at 1 rad/s)	82,037 (± 84,967)	64,556 (± 64,832)	0.0161	41,073 (± 45,478)	0.0009	24,741 (± 35,553)	0.0110
G″ (at 1 rad/s)	20,926 (± 20,403)	16,557 (± 16,337)	0.0597	10,854 (± 11,264)	0.0029	6480 (± 8756)	0.0312
tanδ (at 1 rad/s)	0.2647 (± 0.0426)	0.2665 (± 0.0596)	0.6548	0.3163 (± 0.1631)	0.0049	0.3051 (± 0.0999)	0.5052
G′ (at 10 rad/s)	101,422 (± 109,431)	80,410 (± 83,619)	0.0122	49,784 (± 54,697)	0.0013	47,712 (± 45,942)	0.0223
G″ (at 10 rad/s)	23,882 (± 25,490)	18,108 (± 18,133)	0.0467	11,963 (± 12,200)	0.0051	12,135 (± 10,962)	0.0799
tanδ (at 10 rad/s)	0.2391 (± 0.0479)	0.2575 (± 0.0817)	0.7346	0.3172 (0.1754)	0.0015	0.2823 (± 0.0504)	0.4369

	n = 16	n = 16		n = 14		n = 9	
*For highly elastic sample (G*′ > *100 000 Pa)*							
G′ (at 1 rad/s)	161,725 (± 95,700)	49,150 (± 47,894)	< 0.0001	28,770 (± 34,739)	< 0.0001	35,445 (± 33,747)	< 0.0001
G″ (at 1 rad/s)	39,393 (± 27,405)	12,338 (± 12,035)	< 0.0001	6823 (± 7914)	< 0.0001	8614 (± 7765)	< 0.0001
tanδ (at 1 rad/s)	0.2393 (± 0.0351)	0.2580 (± 0.0727)	0.9311	0.3104 (± 0.1853)	0.1883	0.2875 (± 0.0841)	0.5394
G′ (at 10 rad/s)	204,250 (± 123,409)	60,679 (± 61,262)	< 0.0001	35,913 (± 43,801)	< 0.0001	46,762 (± 42,154)	< 0.0001
G″ (at 10 rad/s)	46,568 (± 35,515)	13,706 (± 13,323)	0.0002	8099 (± 9650)	< 0.0001	10,937 (± 9471)	0.0002
tanδ (at 10 rad/s)	0.2192 (± 0.0312)	0.2431 (± 0.0895)	0.8880	0.3259 (± 0.2016)	0.0367	0.2665 (± 0.0522)	0.5963

	n = 34	n = 34		n = 28		n = 22	
*For elastic sample (100 000 Pa* > *G*′ > *1000 Pa)*							
G′ (at 1 rad/s)	33,999 (± 23,742)	18,133 (± 21,186)	0.0395	17,151 (± 23,067)	0.0398	27,299 (± 38,056)	0.6779
G″ (at 1 rad/s)	8435 (± 5620)	4919 (± 5606)	0.0943	4664 (± 6284)	0.0902	7122 (± 10,032)	0.8262
tanδ (at 1 rad/s)	0.2549 (± 0.0355)	0.2936 (± 0.0536)	0.1204	0.3377 (± 0.1364)	0.0003	0.2949 (0.0671)	0.1623
G′ (at 10 rad/s)	40,702 (± 28,571)	21,513 (± 25,010)	0.0486	20,935 (± 27,391)	0.0583	35,549 (± 50,749)	0.8957
G″ (at 10 rad/s)	9585 (± 6505)	5303 (± 5905)	0.0783	5298 (± 6824)	0.1046	8913 (± 12,723)	0.9811
tanδ (at 10 rad/s)	0.2421 (± 0.0379)	0.2683 (0.0630)	0.7026	0.3427 (± 0.2224)	0.0040	0.2962 (± 0.0835)	0.2315

	n = 14	n = 14		n = 12		n = 6	
*For viscoelastic sample (1000 Pa* > *G*′*)*							
G′ (at 1 rad/s)	272.3 (± 416.7)	368.7 (± 864.8)	0.9746	168.7 (± 268.6)	0.9660	729.7 (± 1031.5)	0.3555
G″ (at 1 rad/s)	117.2 (± 200.1)	129.4 (± 320.2)	0.4434	60.7 (± 109.5)	0.9831	183.6 (± 260.8)	0.9834
tanδ (at 1 rad/s)	0.3885 (± 0.0906)	0.3550 (± 0.1660)	0.9032	0.3815 (± 0.0986)	0.9988	0.3333 (± 0.2049)	0.7357
G′ (at 10 rad/s)	198.2 (± 246.7)	415.7 (± 965.8)	0.8411	217.2 (± 349.0)	0.9999	852.7 (± 1338.6)	0.1843
G″ (at 10 rad/s)	74.2 (± 100.8)	149.9 (± 366.57)	0.8290	74.2 (± 136.1)	> 0.9999	226.9 (± 377.3)	0.4675
tanδ (at 10 rad/s)	0.3446 (± 0.1088)	0.3545 (± 0.2431)	0.9991	0.3662 (± 0.2928)	0.9907	0.3766 (± 0.1579)	0.9818

**Table 2:** Rheological parameters of the sputum using a sputum: additive ratio of 10:1

	Untreated	Water		Bicarbonate		NaCl	
	Mean (SD)	Mean (SD)	p value	Mean (SD)	p value	Mean (SD)	p value
*For all samples*							
G′ (at 1 rad/s)	67,359 (± 88,421)	67,175 (± 66,877)	> 0.9999	57,423 (± 73,261)	0.9789	126,093 (± 218,382)	0.9996
G″ (at 1 rad/s)	16,900 (± 20,838)	16,808 (± 17,012)	> 0.9999	14,590 (± 17,772)	0.9822	32,291 (± 55,925)	> 0.9999
tanδ (at 1 rad/s)	0.2654 (± 0.0400)	0.2483 (± 0.0359)	0.7877	0.2700 (± 0.0399)	0.9936	0.3623 (± 0.2026)	0.1841
G′ (at 10 rad/s)	85,329 (± 114,754)	83,220 (± 86,399)	> 0.9999	71,183 (± 87,859)	0.9697	82,689 (± 128,089)	0.9999
G″ (at 10 rad/s)	20,040 (± 26,749)	18,140 (± 18,996)	0.9932	16,429 (± 19,110)	0.9576	19,947 (± 29,187)	> 0.9999
tanδ (at 10 rad/s)	0.2444 (± 0.0497)	0.2325 (± 0.0447)	0.8464	0.2565 (± 0.0465)	0.8355	0.2669 (± 0.0481)	0.5015

	n = 4	n = 4		n = 4		n = 3	
*For highly elastic sample (G*′ > *100 000 Pa)*							
G′ (at 1 rad/s)	183,109 (± 94,456)	111,662 (± 75,718)	0.3810	74,551 (± 50,395)	0.1225	45,154 (± 34,271)	0.0641
G″ (at 1 rad/s)	43,568 (± 21,332)	27,415 (± 21,453)	0.4412	18,263 (± 11,377)	0.1459	10,924 (± 7285)	0.0745
tanδ (at 1 rad/s)	0.2432 (± 0.0299)	0.2272 (± 0.0362)	0.8908	0.2502 (± 0.0315)	0.9886	0.2526 (± 0.0595)	0.9786
G′ (at 10 rad/s)	236,912 (± 122,817)	145,972 (± 103,271)	0.3999	93,302 (± 60,342)	0.1168	58,010 (± 43,427)	0.0659
G″ (at 10 rad/s)	54,340 (± 29,671)	32,120 (± 24,897)	0.3797	20,330 (± 13,121)	0.1185	13,729 (± 9456)	0.0803
tanδ (at 10 rad/s)	0.2283 (± 0.0243)	0.2075 (± 0.0246)	0.6740	0.2178 (± 0.0061)	0.9320	0.2420 (± 0.0578)	0.8889

	n = 7	n = 7		n = 7		n = 6	
*For elastic sample (100 000 Pa* > *G*′ > *1000 Pa)*							
G′ (at 1 rad/s)	33,999 (± 23,742)	18,133 (± 21,186)	0.5433	17,151 (± 23,067)	0.9883	27,299 (± 38,056)	0.7230
G″ (at 1 rad/s)	8435 (± 5620)	4919 (± 5606)	0.6247	4664 (± 6284)	0.9900	7122 (± 10,032)	0.5802
tanδ (at 1 rad/s)	0.2549 (± 0.0355)	0.2936 (± 0.0535)	> 0.9999	0.3427 (± 0.2224)	0.3416	0.2949 (± 0.0671)	0.7706
G′ (at 10 rad/s)	40,702 (± 28,571)	21,513 (± 25,010)	0.5474	20,935 (± 27,391)	0.9925	35,549 (± 50,749)	0.8276
G″ (at 10 rad/s)	9585 (± 6505)	5303 (± 5905)	0.6678	5298 (± 6824)	0.9989	8913 (± 12,723)	0.7473
tanδ (at 10 rad/s)	0.2421 (± 0.0379)	0.2683 (± 0.0631)	0.7958	0.3427 (± 0.2224)	0.3426	0.2962 (± 0.0835)	0.2537

	n = 5	n = 5		n = 4		n = 4	
*For viscoelastic sample (1000 Pa* > *G*′*)*							
G′ (at 1 rad/s)	1453.5 (± 2831.3)	1104.9 (± 1875.1)	0.9921	1306.7 (± 1502.5)	0.9994	10.3 (± 13.3)	0.7975
G″ (at 1 rad/s)	438.8 (± 854.2)	360.2 (± 614.7)	0.9968	393.6 (± 456.2)	0.9994	2.6 (± 2.9)	0.8069
tanδ (at 1 rad/s)	0.2880 (± 0.0269)	0.2623 (± 0.0582)	0.9696	0.2783 (± 0.0368)	0.5187	0.4155 (± 0.2553)	0.9982
G′ (at 10 rad/s)	2309.5 (± 4540.5)	682.8 (± 1132.2)	0.8451	2011.5 (± 2365.9)	0.9986	528.1 (± 709.9)	0.8572
G″ (at 10 rad/s)	698.7 (± 1374.1)	238.0 (± 400.1)	0.8670	557.7 (± 675.9)	0.9948	166.2 (± 226.0)	0.8607
tanδ (at 10 rad/s)	0.2923 (± 0.0142)	0.2743 (± 0.0677)	0.9020	0.2797 (± 0.0222)	0.9614	0.2790 (± 0.0523)	0.9686

When a large volume of additive (10:4, Table 1, Box plots of the results are presented in Additional file 1: Figures S-1–4) was applied, the viscoelastic moduli (G′ and G″) decreased, while the loss factor (tanδ) increased, indicating the destruction of the highly elastic gel structure. Only the hypertonic NaHCO_3_ solution caused significant changes in all rheological parameters, whereas distilled water and hypertonic NaCl solution did not change the loss factor values.

When the results were evaluated by category, all additives significantly decreased the rheological parameters of the highly elastic and elastic sputum samples (with the exception of the loss factor). However, the most effective additive was bicarbonate (the lowest G′ and G″ values).

For elastic and viscoelastic samples, where the sputum had a more liquid state, the additives did not induce significant changes in the rheological parameters, only in the case of bicarbonate or water we could find significant change in some parameters (G′ and loss factor).

When using lower volumes of additives (10:1, Table 2), we observed a tendency for decreased values in the rheological parameters (especially using NaHCO_3_ or NaCl solutions for highly elastic sputum samples). However, because of the variability of the samples, the differences were not statistically significant at this mixing ratio (Table 2, Box plots of the results are presented in Additional file 1: Figures S-5–8).

## Discussion

Several previous studies have investigated the rheological properties of CF sputum samples and shown that they have viscoelastic behaviour, which can be studied by oscillatory rheometry (microbead rheology or shear rheology) [8]. Nonetheless, rheological measurements of sputum samples face a number of challenges. Samples from spontaneous expectoration are normally diluted by saliva, thus pre-separation is required to assess the properties of the sputum itself. Sputum samples may exhibit significant variability, even from the same individual. There are many possible explanations for this, for example colonization with different bacteria, differences in ASL composition, various degrees of contamination by saliva [14, 15], and different intensities or frequencies of repetitive voluntary cough. Any of these factors can alter the solid content and the hydration level of the sputum and thus its viscoelastic properties [16]. The small sample size and heterogeneity within the sample can also complicate the analysis.

In the present study, we investigated some of the methodological factors influencing the rheological measurements. Sputum samples were separated from saliva by using the centrifugation technique described previously in the literature [4, 17]. After separation, the sputum gel phase was gently homogenized by stirring. For rheological analysis, a resting measurement phase was introduced to investigate whether pre-homogenization, the mechanical effects of sample agitation and insertion into the rheometer, or storage temperature could affect the rheological parameters and their change over time. Our results clearly demonstrate that the introduction of the resting phase into the measurement protocol is recommended because sputum samples undergo significant changes once inserted into the rheometer, which could be due to the unavoidable but disruptive effects of sample operation. The length of this section can be optimized for a given instrument and preparatory operation. It should be long enough for stabilization of the parameters. However, efforts should be made to limit the preparation time as short as possible to avoid the dehydration and degradation of the samples. In our experiments we established this time period of 30 min.

The technique used to mix the sputum with additives, such as mucolytics, can significantly alter the initial rheological properties of the sample. For example, the gel structure was broken down after ultrasonication and did not recover during the test stage. In contrast, pre-warming the samples did not appear to change the gel structure.

The rheological properties of sputum can be influenced by a number of factors, such as solid content, the degree of hydration, pH, electrolyte concentrations, the quantity and types of mucins, extracellular materials (e.g. extracellular DNA and F-actin), and interactions between mucin chains or with other molecules [18]. Mucolytic agents mainly target these interactions, but they may also influence the pH and/or hydration state of the ASL.

The use of hypertonic saline (HS) solution is an inexpensive, safe, and effective additional therapy in CF patients with stable lung function [19]. The inhalation of HS solution can improve mucociliary clearance due to its hyperosmolarity, where water transport into the airways is driven by osmosis, resulting in a deeper periciliary fluid layer and enhanced mucus hydration [20, 21]. Elkins and co-workers reported significant benefits of hypertonic saline inhalation in a 48‐week parallel group study, in which 164 CF patients were randomised to receive either 7% HS (300 mmol/L NaCl) or placebo (150 mmol/L NaCl) [21]. It was demonstrated that FEV_1_ was approximately 3% higher in the HS than in the placebo group. The effects of other osmotic agents, mannitol [22] and xylitol [23, 24], have also been studied in CF patients.

The inhalation of bicarbonate-containing solutions could be another useful adjuvant therapy in CF. NaHCO_3_ is an effective, safe and well-tolerated therapeutic agent in CF and possibly in other chronically infected lung diseases [7, 25, 26]. Gomez et al. have recently demonstrated that the inhalation of hypertonic NaHCO_3_ increased the pH of the ASL and decreased the gel strength of the sputum, which could be explained by greater expansion of mucins and DNA. This weakening effect of bicarbonate on gel strength has also been reported by Stigliani et al. [4] who showed that elastic and viscous moduli, as well as complex viscosity, were reduced after in vitro treatment. This group has also investigated the dissolution and permeation properties of ketoprofen lysinate (Klys) in CF sputum. Interestingly, CF sputum treated with NaHCO_3_ exhibited more rapid Klys dissolution and permeation than sputum without added bicarbonate [4].

In our study, the effects of NaCl and NaHCO_3_ solutions (300 mmol/L) were investigated on CF sputum samples in vitro. As a reference treatment, water was added to some sputum samples, which can be regarded as an indicator of the hydrating effect of the additive solution without the salt or bicarbonate. The overall aim of the present work was to develop an in vitro method to predict the efficacy of topically applied mucolytics. This could help in selecting the most appropriate inhalation formulation for the disease status of each CF patient. However, it must be kept in mind that the method presented here is not suitable for assessing the beneficial in vivo osmotic effects of hypertonic solutions.

Storage and loss modulus as well as the loss factor can be used to compare the efficacy of different in vitro treatments. The gel structure and its viscoelastic characteristics can be well characterized by measuring these rheological parameters. Mucus clearance is known to be influenced by viscoelasticity [5]. Two main mucus clearance processes can be distinguished: (i) mucociliary clearance (MCC) and (ii) cough clearance (CC). The efficacy of these clearance processes depends upon the rheological properties of sputum. In previous studies, MCC was simulated by using low frequency deformations (1 rad/s), while CC was mimicked by applying 100 rad/s. In micro-rheology, these frequencies correspond to the beating frequency of epithelial cilia and coughing, respectively [5, 27]. In shear rheology, it has been suggested that higher frequencies should be avoided because the inertia of the instrument strongly affects the torque response of the soft CF sputum. Since it has been recommended to limit frequencies to no higher than 10 rad/s [14, 28], we compared the rheological parameters (G′, G″ and tanδ) of the untreated and treated sputum at 1 and 10 rad/s.

The sputum samples showed great variability based on the elastic modulus at 1 rad/s of the untreated samples. The range of these elastic moduli correspond to values described in a previous study [4] in which the same sputum preparation method was applied. Importantly, we detected 3 distinct categories: (i) highly elastic (G′ > 100,000 Pa), (ii) elastic (100,000 Pa > G′ > 1000 Pa), and (iii) viscoelastic (G′ < 1000) sputum samples. Visually the highly elastic samples were very compact and behaved as a solid material. Elastic samples showed remarkable elasticity, but they were deformable, while viscoelastic samples presented liquid characteristics. The changes in the rheological parameters, with time and additive pre-treatment, were therefore assessed in each category separately.

In this study, we did not assess the clinical status of the patients, thus the conclusions were drawn on the basis of the rheological properties of the sputum samples alone. They were treated either at 10:4 (n = 61) or 10:1 (n = 15) sputum/additive solution ratios. We used a ratio of 10:1 (sputum/additive) based on methods published by Stigliani et al. [4]. In addition, we have chosen a ratio of 10:4 which may mimic the luminal environment following application of the hypertonic saline and water secretion.

When using the larger volume of additive (10:4), the gel strength of the sputum decreased because the viscoelastic moduli decreased and the loss factor increased at each frequency. The pronounced variability of the samples is reflected in the large standard deviation values, which are even more noticeable in the treated samples. Importantly, we found that the added solutions significantly reduced the gel strength of the sputum considering all investigated samples, but the most pronounced changes (lowest p values) were observed for NaHCO_3_ (p < 0.001). Samples with high elasticity (G′ > 100,000 Pa) exhibited the greatest changes in the parameters, suggesting that dilution of the gel structure may result in the greatest structural breakdown in samples of this type. It is remarkable that even more significant effects were detected when the samples were treated by NaHCO_3_.

For less elastic samples, a weakening of the gel structure was also observed when they were treated with distilled water or NaHCO_3_, but not with NaCl solution, where there were no significant changes in the parameters. For viscoelastic samples with a low elastic content, the additives did not cause significant changes in the parameters.

For all samples at 10:4 ratio, the effect of additives significantly reduces the rheological parameters of the sputum, but when the changes within the categories are analysed, it is clear that the reducing effect is mostly limited to samples with significant elasticity (highly elastic and elastic samples), at low elasticity no significant effect can be observed.

When a lower additive volume (10:1 ratio) was used, the mean values of the rheological parameters usually decreased, especially in the case of the highly elastic sputum samples following treatment with either NaHCO_3_ or NaCl solutions. A decrease in modulus was also observed in this additive ratio. The effects of NaCl were the most remarkable (but not significant) in the most elastic samples, while the efficacy of NaHCO_3_ was highest in the middle category (lower mean values observed in the case of the treated samples, in Table 2). The beneficial effects of NaCl could also be observed in the sputum samples with the lowest elastic content (G′ < 1000 Pa). Considering all samples treated with 10:1 sputum to additive ratio, the bicarbonate treated samples showed the lowest mean rheological parameters (and thus the more effective treatment), but within the categories this tendency is not prevailed, the effect of additives is different in the case of samples with different rheological profiles. However, it should be noted that no significance was detected in this sputum/additive ratio.

When comparing the alternative sputum/additive ratios used here, it is evident that larger amounts of added water alone can significantly reduce the gel character of the sputum samples. Based on the rheological properties of the initial sputum samples, it may be appropriate to categorize the treatment efficacy by each additive. The difference between the categories suggests that it is advisable to evaluate each sample individually. These efficacy measurements in vitro may be suitable for such an assessment.

In accordance with previously published observations, the standard deviations of the mean values were also high in the present work, which rendered the statistical analysis of the data difficult. However, the introduction of the resting phase, or the equilibration of the samples immediately before measurements may reduce the inaccuracy of the data.

## Conclusion

In this study we have assessed and compared in vitro the effects of hypertonic mucolytics that are used locally as inhaled aerosols for adjuvant therapy in CF. Our data suggest that the pre-treatment and handling of sputum samples can exert significant effects on their rheological properties. Thus, it is important to investigate the adverse effects of sample treatment in all applied rheological methods. In assessing the effects of hypertonic NaCl and NaHCO_3_ solutions on CF sputum samples, we observed that these mucolytic agents exhibit different effects on sputum samples according to their initial viscoelastic characteristics. Our results suggest that it is advisable to evaluate each sample individually, and our measurements for the analysis of the efficacy of the additives in vitro may be suitable for such an assessment.

## Supplementary Information

**Additional file 1.** Box plot of rheological parameters of the sputum samples.

## Data Availability

The datasets used and/or analysed during the current study are available from the corresponding author on reasonable request.

## Abbreviations

CF: Cystic fibrosis
CFTR: Cystic fibrosis transmembrane conductance regulator
G′: Storage modulus
G″: Loss modulus
tanδ: Loss factor
ENaC: Epithelial Na^+^ channel
ASL: Airway surface liquid
MCC: Mucociliary clearance
CC: Cough clearance
HS: Hypertonic saline
FEV_1_: Forced expiratory volume, the amount of air forced from the lungs in one second
