# Supplementary material for: Rheological effects of hypertonic saline and sodium bicarbonate solutions on cystic fibrosis sputum in vitro
Source: BMC Pulm Med. 2021 Jul 12;21:225. doi: 10.1186/s12890-021-01599-z (PMC8276516; doi:10.1186/s12890-021-01599-z)
Supplement: Supplementary file 1 — Additional file 1. Box plot of rheological parameters of the sputum samples. [file 12890_2021_1599_MOESM1_ESM.docx]

S-1 Figure Box plot of rheological parameters of the all sputum samples using a sputum additive ratio of 10:4, where a,c and e represent data at 1 rad/s; and b, d and f represent data at 10 rad/s. (*, **, and *** indicate significant, very and highly significant, respectively.)

S-2 Figure Box plot of rheological parameters of the highly elastic sputum using a sputum additive ratio of 10:4, where a,c and e represent data at 1 rad/s; and b, d and f represent data at 10 rad/s. (*, **, ***, and **** indicate significant, very, highly, and very highly significant, respectively.)

S-3 Figure Box plot of rheological parameters of the elastic sputum using a sputum additive ratio of 10:4, where a,c and e represent data at 1 rad/s; and b, d and f represent data at 10 rad/s. (*, **, ***, and **** indicate significant, very, and highly significant, respectively.)

S-4 Figure Box plot of rheological parameters of the viscoelastic sputum using a sputum additive ratio of 10:4, where a,c and e represent data at 1 rad/s; and b, d and f represent data at 10 rad/s.

S-5 Figure Box plot of rheological parameters of the all sputum samples using a sputum additive ratio of 10:1, where a,c and e represent data at 1 rad/s; and b, d and f represent data at 10 rad/s.

S-6 Figure Box plot of rheological parameters of the highly elastic sputum using a sputum additive ratio of 10:1, where a,c and e represent data at 1 rad/s; and b, d and f represent data at 10 rad/s.

S-7 Figure Box plot of rheological parameters of the elastic sputum using a sputum additive ratio of 10:1, where a,c and e represent data at 1 rad/s; and b, d and f represent data at 10 rad/s.

S-8 Figure Box plot of rheological parameters of the viscoelastic sputum using a sputum additive ratio of 10:1, where a,c and e represent data at 1 rad/s; and b, d and f represent data at 10 rad/s.
